# Using data to make the case for program resources and sustainability: the BEST action inventory case study

**DOI:** 10.1007/s43545-021-00137-2

**Published:** 2021-05-17

**Authors:** Julie M. W. Rojewski, Nadia Ayala-Lopez, Sean Nguyen, Stephanie W. Watts

**Affiliations:** 1 MSU Graduate School, Michigan State University, 466 W. Circle Drive, Room 130C, East Lansing, MI 48824-1317 USA; 2 Johns Hopkins University School of Medicine, Baltimore, MD USA; 3 Cell and Molecular Biology Program, Michigan State University, East Lansing, MI USA; 4 Department of Pharmacology and Toxicology, Michigan State University, East Lansing, MI USA

**Keywords:** Graduate students, Post doctoral, Biomedical, Career and professional development, Assessment, Program review

## Abstract

Career development programs are a valuable part of any student’s experience, and increasingly is an expected part of graduate school training. While such programs are commonly available to undergraduates, there is a growing need for career support to be offered to graduate students. Making the case for resources can be a challenge in this domain, however. Research on the impact of career services for graduate students and post-doctoral scholars is a growing scholarly concern. However, there remains a need to better understand what level of intervention is most appropriate: What kind of activities, how much time, and what resources would best serve the professional development needs of graduate students and post-doctoral scholars? And to answer these questions, a more foundational one: what activities are drawing the attention of graduate students and post doctoral trainees, and in what activities are they spending their time? In this manuscript, we describe how Our University approached this research question by developing an online data tracking system to capture graduate and post-doctoral trainee participation in one co-curricular professional development program. We demonstrate how this data tracking system can be used to advocate for institutional resources in career development programming, for research, and for practical purposes such as advocating for institutional support and for program design and assessment.

## Introduction

Higher education scholars and administrative leaders are increasingly preoccupied by the changing career landscape for students. Furthermore, the social and economic effects of COVID-19 explicitly challenged foundational beliefs about post-secondary education (Arum and Stevens 2020; Nadworny 2020) and the stability of the future of higher education (Kelsky 2020; Wood 2020). Students and others question whether graduate degrees, in particular, are “worth it” (Bledsoe and Oatsvall 2009; Hogan and Ramamurthy 2020; Okahana and Hao 2019) among a job landscape that remains uncertain (Carey 2020). As educators and students grapple with confronting the “why” of graduate education, a related debate emerges: is a degree a credential, a form of job training, continued learning, evidence of expertise, or does it serve a completely different function? (Morris 2017; Tomlinson 2012). When we settle on the “value” of degrees, how do we capture and report these values (Gibbs and Griffin 2013; Goldberg et al. 2016) to potential employers, to policymakers and institutional leaders considering what to fund and at what levels, or to students who may be expected to fund their degree program?

This is not a new problem; such questions have preoccupied those working in undergraduate education for years. Graduate schools and graduate students have begun asking similar questions, prompted by changes in a job market for advanced degree holders. The job market no longer leads singularly to positions in academia. Rather, “traditional” academic careers are available to a minority of doctoral degree earners within a broader career landscape for Ph.D.s (Cassuto 2015; Hankel 2019). Many hiring managers outside academia value the maturity and problem solving skills Ph.D.s bring to a variety of jobs (Chhinzer and Russo 2018).

Many institutions, then, are actively trying to address job placement uncertainty and the degree of “worth” in attaining an advanced degree. In 2019, the Association of American Universities (AAU) announced a pilot program called the Ph.D. Education Initiative “to promote more student-centered doctoral education at AAU universities by making diverse PhD career pathways visible, valued, and viable” (AAU 2019). Professional organizations, such as the Graduate Career Consortium, emerged to share research and strategies to better serve the specific career needs of graduate students and postdocs across varied disciplines. Institutions are finding ways to support their students and consider how to expand upon a traditional undergraduate “career services model” to better support graduate students (Patrick 2011; Ritter et al. 2018), while also addressing the specific challenges of graduate students as they transition to the workplace (Evans et al. 2018).

Scholarship that looks more closely at professional development in the graduate space is also growing. Collectively, we as education leaders realize the important responsibility to equip graduate students for entrance into a rapidly-changing, and often uncertain, career landscape.

### The need for data to facilitate evidence-based decisions

Recognizing a need for action but unclear where to begin efforts in the absence of evidence-based protocols, scholars and administrators require data to help identify needs and to begin planning how to allocate resources (Soares et al. 2016). When it comes to research around professional development, there remain questions about what data to capture and *how* to capture these data. In this study, we illustrate how one graduate career and professional development program at our university addressed this issue. We developed an online data-tracking system that aggregates trainee engagement in career-development-activities. We built the online data-tracking system to provide formative assessment, capture usage patterns, and yield foundational data that can be used in longitudinal research to assess outcomes as participants advance in their careers.

Here, we describe our online data-tracking system to provide data on professional development activities that can improve trainee career preparation. The ability to capture professional development participation offers additional insight into the activities that students may have believed had the most value for their future career and to use that information to boost engagement by designing future programs with their specific preferences in mind. We also discuss how an online data-tracking system could support initiatives to increase institutional investment into resources that support career development of graduate level learners.

### Review of the literature

There is a recognized value to studying the rollout of these efforts, moving career development programming beyond undergraduate students to include graduate students and postdoctoral scholars (Allais 2012; Heflinger and Doykos 2016; Jackson and Collings 2018; Werquin 2012). This move toward seeing degree pursuit as career training for people at all levels of education and across diverse fields of study reflects changes in the job market (Gemme and Gingras 2012; Solem et al. 2013) for Ph.D.s, as well as reflects an overall shift to thinking about post-secondary education as training *for* jobs.

Organizations are making explicit the connection between college (or advanced) degrees as evidence of learning and knowledge, as well as a part of a career strategy to help students attain the jobs they seek. Consider the 2016 report from the Association of American Colleges and Universities, *Trends in Learning Outcomes Assessment*. In this report, AAC&U President Carol Geary Schneider calls for ‘specific cross-cutting capacities or learning outcomes [that] are absolutely necessary for any graduate who wants to succeed in today’s economy and in a complex, fast-changing world,’ (Hart 2016). Such competencies (Surr and Redding 2017), directed at ‘succeeding in today’s economy,’ include critical-thinking, problem solving, communications, integrated learning, and other transferable skills (Kemp and Seagraves 1995). These skills are also identified by employers as something they seek in potential employees (Matthews and Mercer-Mapstone 2018; Woodside 2018) and are used by Ph.D. students and postdocs in a wide variety of careers.

The pressures put on higher education leaders to respond to these dual influences–changing job markets for students, and a belief that education should be linked to career outcomes—compels institutions to offer necessary support for their students to achieve professional and career-related goals (Cole et al. 2018). University leaders are tasked with delivering opportunities that help support students across their educational lifespan, which includes adapting resources traditionally offered to undergraduates to be suitable for graduate students as well.

In so doing, education leaders must determine how best to go about building coalitions that provide adequate resources for the “right” amount of professional development for students. Often, making such a case relies upon having the right data. Colleges and universities are increasingly data-driven institutions, challenged by financial pressure and calls for “accountability” that show evidence of success and impact. An emphasis on “big data and analytics” (Catalano 2019; Picciano 2012) has inspired scholars and practitioners to find ways to try to advocate for resources and show evidence of impact.

Scholarship and research on assessment and program evaluation of career preparation programs predominantly studies undergraduate populations (August et al. 2010; Grier-Reed and Chahla 2015; Hansen and Pedersen 2012), though there is a growing body of scholarship that focuses on assessments of and for graduate students (Helm et al. 2012; Holaday et al. 2007; Ortega and Kent 2018). This shift is likely explained in part by shifting career landscapes (McAlpine and Austin 2018) and aspirations (Jones et al. 2012; Solem et al. 2013; Wilson et al. 2018; Woodside 2018) among students pursuing advanced degrees.

The technology and emerging scholarship in the domain of “comprehensive learner record” (CLR) is an example of how colleges and universities seek to capture engagement in co-curricular experiences (IMS Global Learning Consortium 2017). “Some institutions have sufficient internal capacity to create the infrastructure that a CLR requires, while others may elect to work with outside vendors and an external data warehouse” (Educause 2019). Implementations of and studies about CLRs are expanding rapidly, in part due to funding from the Lumina Foundation grant that supports a joint effort of the American Association of Collegiate Registrars and Admissions Officers, (AACRAO) and the National Association of Student Personnel Administrators (NASPA) (Educause 2019), who are trying to streamline and scale up these efforts. Students, employers, and institutional leaders see this as a formal record of student engagement beyond a traditional transcript that can also capture leadership, service learning opportunities, membership in clubs and student organizations, and other activities. In fact, two years after the launch of the study we describe here, Michigan State University (MSU) announced the launch of a CLR on our campus targeted at undergraduate students.

CLRS are growing in popularity and research on their impact is certain to follow. It is unclear if this research will focus on CLRs as a technology that primarily serves undergraduates or if it will also include graduate students. Regardless, this growing body of research shows a commitment to better understanding the availability and impact of co-curricular experiences on graduate students and post-doctoral fellows, which is the focus of the present study.

Other relevant graduate-centered scholarship explores the relationship between graduate education, graduate training, and career exploration (Knudson, Gutstein, and Evans 2011), changing attitudes about the value of doctoral education (Bryan and Guccione 2018; Hum 2015; Schnoes et al. 2018), and factors inspiring new Ph.D.s to investigate careers outside academia (Dorenkamp and Weiß 2018; Gibbs and Griffin 2013). Institutions, funding agencies, and other organizations are redoubling their efforts to support graduate students interested in broad careers (National Institutes of Health 2012) and more people working in graduate education at all levels are interested in capturing trends in programming and evaluating the effects of such interventions (Rizzolo et al. 2016).

This growing area of research will shape institutional efforts to support graduate students and their co-curricular career preparation and help institutional leaders make the case for resources to be used in the service of these goals.

The need for such programs—explicit connections between learning/educational outcomes and career readiness—has been articulated, and scholarship and evaluation efforts continue to assess impact. Collectively, the scholarship and research evidence also justify investment in career and professional development for graduate students and continued study about what works for this population. Still, in our efforts to build, assess, and sustain a new career and professional development program at MSU, we found one challenge to be the need for *participation* data specific to *our* institution and about *our* students that would help make our case to institutional leaders. In response, we developed a data-tracking system whose utility proved effective for making our case and, we believe, can be useful for other programs interested in tracking metrics in similar ways.

Our study seeks to accomplish two goals. First, to inform our research question: how much and what type of professional development activity seem to be most appealing to students in pursuing their career goals? And what doses of intervention will eventually prove most effective? The latter question is one we are tracking through longitudinal data collection: when a larger number of students in this population graduate and move on to the next phase of their training, we will be able to go back to the data we collect *now* to identify patterns of participation and attention. In this paper, we introduce early data that we have found useful for the first question and provide the foundation for longitudinal analysis. We also believe that this system for capturing participation data has immediate applications for practitioners in higher education.

## Theoretical framework

We describe throughout the methods section of this paper the development of the tool, and the technical aspects of its utility as a data collection instrument. We introduce “minutes” as the unit of analysis, which seems almost old-fashioned in its simplicity. After all, merely participating in any sort of training activity is insufficient to assess its utility or impact: engagement is, arguably, a more aspirational goal and as educational researchers, we share a commitment to ensuring quality by assessing outcomes, behavioral change, and other metrics that do a better job than “minutes” in assessing quality (Kuh et al 2014; Makela and Rooney 2012; Suskie 2009). Indeed, we also incorporated such assessment strategies to assess program-level outcomes for the project described here. Concurrent and complementary to such analysis, and for the purposes of the analysis described in this paper, however we needed a simpler form of data: simple participation.

That is the focus of this paper, and there are several reasons why we believe that these data have utility to a program assessment like this.

First was the need to establish and agree upon vocabulary to ensure that we could measure what we needed to measure, which in this case was to capture both *participation* as it happened, *engagement* when we could assess our own programming, and *impact* in the future. Ashwin and McVitty (2015) note “Student engagement has come to be seen as a ‘good thing’ in higher education for researchers and policy makers alike.” However, the definition of what engagement is and what it does has grown more expansive, but less clear (Ashwin and McVitty 2015; Reschly and Christenson 2012). It can refer to participation in activities in which the student is “psychologically invested” and takes “pride” in their work (Newman et al. 1992), activities in which they are “focused” and “invested” (Schlechty 2011), or activities which arouse “enthusiasm” in students (Frensley et al. 2020) and a variety of “kinds” of engagement (Park et al. 2012). As a research and program team, we aimed to offer engaging programming, but at every point realized the need to study not only engagement, but also participation. We wanted to explore different locations of professional development, different types and durations, and frequency of participation. We wanted to know if and how trainees were spending their time and attention in matters of career and professional development.

Further, many models of and scholarship about “engagement” focus on K-12, or even K-16 contexts; often with a focus on defined school-related environments and programming (Lawson and Lawson 2013). As we noted earlier in this piece, scholarship is growing around the proliferation and impact of graduate student and post-doctoral trainee (post-doc) professional development. However, co-curricular professional development is not a given for every graduate student or post-doc in every program, and for some, the mere act of *participating* in a co-curricular professional development opportunity can be a revolutionary act, one that empowers individual graduate students and post-docs to find information or support beyond one’s own mentor or training program (Benderly 2017).

For others, a full embrace of all kinds of professional development is welcomed, even encouraged. We are interested in how students are spending their time and capturing the participation patterns of students who may only be starting to venture into professional development as well as those who seek out and engage fully in professional development of all types and multiple levels. By focusing on minutes as a unit of measure, we are able to capture the range of student participation, be it tentative or wholehearted, be it passive attendance at an online panel or full-embrace of an internship that may take someone off campus for a longer period of time. The level of engagement across this spectrum varies, but for our study, all minutes matter.

A third reason for our use of “minutes” as a unit of measure is that it afforded us a way to capture professional development participation in activities outside the campus-sponsored programs. Like many offices, our unit routinely assesses the impact of our programs on predetermined learning outcomes. We could easily track attendance to capture participation—and engagement—data at program-sponsored events. However, we recognize that professional development extends beyond these events and can be a highly personalized experience. Thus, to truly understand this process for each of our trainees it was important to capture participation in other offerings: career programming at professional conferences, workshops in an individual unit, online professional development seminars, or conversations over coffee with a guest speaker where career advice is shared. It would be difficult to assess engagement and impact of this wide variety of events; asking trainees to track the number of minutes spent in those events, regardless of where or by whom they were offered, gives us a more comprehensive representation of their specific level of engagement.

The final reason we used minutes as our unit of measure was this: though basic, capturing participation rates and numbers allowed us to identify trends that were useful in improving programming, advocating for resources, and helping us narrow which activities were potentially most robust for future, comprehensive, assessment and analysis. For example, by analyzing participation data alone, we could determine which program topics were most likely to attract participation. This tool allowed us to identify students who were most frequently participating in different activities—whether those activities were ours or offered elsewhere—and target them for specific support and opportunities that supported their interests and engagement. And it allowed us to make the case for resources because we could show which students, from which departments, and at what intervals were taking advantage of opportunities to develop their career preparedness. Participation data enhanced other evidence of impact (such as more comprehensive assessment efforts) but even raw numbers can be valuable when making a case for institutional support. After all, while we are interested in capturing data on the impact of our programming and how it affects participation, we also needed to start with a more basic question: who is coming “through the door?” And, which doors?

**Fig. 1:**
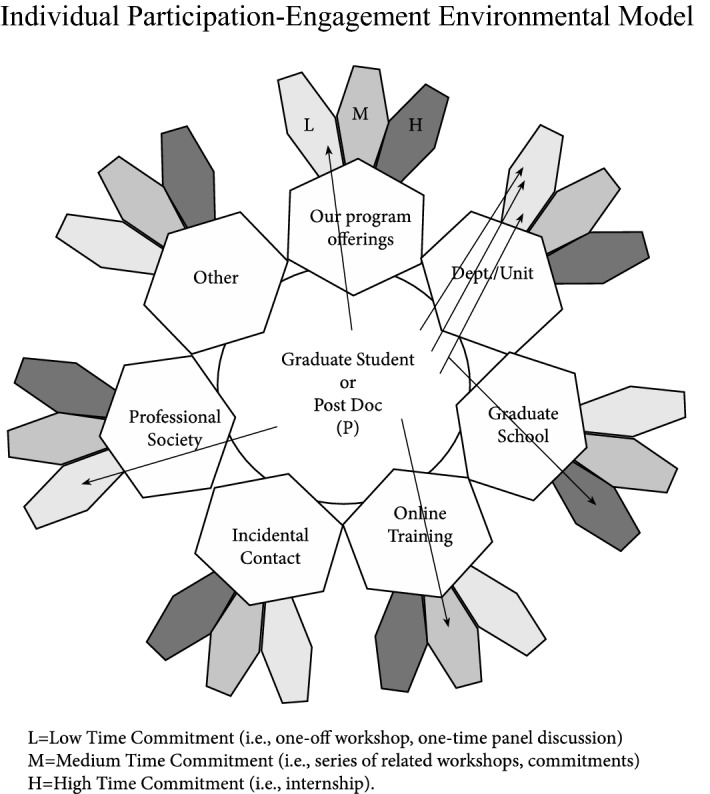
Individual participation-engagement environmental model

**Fig. 2:**
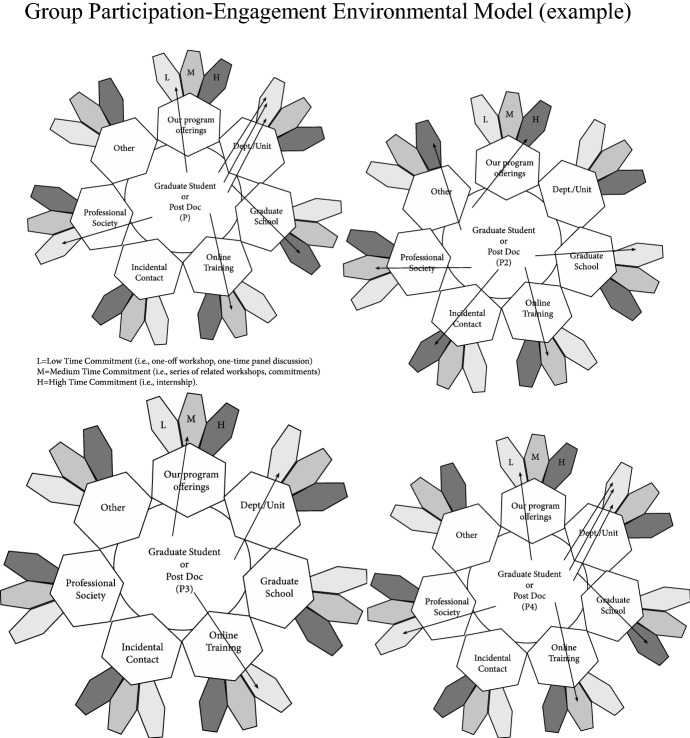
Group participation-engagement environmental model (example)

As scholars grapple with the murky definitions around engagement and build upon work that attempts to see more globally the environments and conditions that support “engagement,” (Lawson and Lawson 2013), we add to this research by building on various models that capture participant activity. We developed a model that echoes some of this work that captures individual (Fig. 1) and comparative (Fig. 2) Participation-Engagement Environments. Our model recognizes that individual participating graduate students and post-docs can and do participate in professional development in a variety of contexts, in addition to the programming we offer them. We encourage such behavior; we also were eager to see if we could identify patterns in how this participation was happening.

Before we could study the impact of these learning opportunities on the students and post-docs we serve, before we could modify our programming to better serve the identified needs of the students in our program, and before we could perform a longitudinal analysis of how this impacted their career goals and outcomes, we needed to address a more basic question: who within our population, how often, how long, and how frequently was “showing up” to any sorts of professional development opportunity? We sought a chance to capture participation data for more than one person, and for activities that were beyond those we offered: we wanted to capture more “arrows” across all of the “flowers” we could (Fig. 2).

And to do that, we developed the BEST Action Inventory tool.

## Methods

In this section, we introduce participants in this study, review the development and launch of the BAI tool, and explain how we sought to maximize the value of the data captured in the system.

### Participants

All of the participants in this study are graduate students and postdoctoral (post docs) trainees from biomedical science and engineering disciplines. Though graduate students and post docs are at different career stages and have differing needs, both populations were eligible to participate in our BEST program; thus we treated them similarly for our program and this analysis. All were members of a cohort within the MSU BEST (Broadening Experiences in Scientific Training) program. In this paper, we refer to them variously as participants, trainees, graduate students, or post docs.

For this study, we had a total sample of 120 students across 4 cohorts. Within the population, there were 65 women (~ 54%) and 55 (~ 46%) men. This is noteworthy because women are underrepresented in their respective graduate programs at our university, and thus overrepresented in BEST, an observation that deserves further attention.

Similarly, of the 120 participants in this study, only 7 (~ 6%) were international trainees, which represents a smaller percentage of international student/postdoc participation than the broader population in biomedical science and engineering. This can be explained by some confusion around international students’ eligibility to apply for BEST: many students believed that because it was funded by a U.S. government funding source (NIH), only U.S. citizens and permanent residents were permitted to join the program.

Of the total population of participants, 22 (~ 18%) self-identified as Hispanic or Latino. When asked to self-identify with a racial category, 4 trainees (~ 3.3%) self-identified as Black, 19 (~ 15.8%) as Asian, and 6 (5%) as more than one race. Eighty-one participants (67.5%) self-identified as White. We offer these demographic data as background information about the participants in this study.

All participants came from a biomedical science or engineering discipline. Each participant applied to the program by submitting an essay, a letter of support from their PI or mentor indicated support for the trainee’s participation in BEST, and a copy of their CV. Upon favorable review by our program staff and internal advisors, participants were admitted to a cohort of “BESTies,” who began the program together at the beginning of an academic year, and participated in a series of activities, including workshops, seminars, guest speakers, and other events.

Though many of those events were specifically coordinated by the BEST program staff, we strongly encouraged BEST trainees to seek out other opportunities, be they at professional conferences, local organizations, in their department or graduate program, at other institutions, or wherever they found an intriguing professional development opportunity. BEST activities were open only to BEST cohort members but BEST cohort members engaged in activities both inside and outside of BEST events.

### Context

This work is part of our campus’s BEST program experiment. Michigan State University was one of 17 BEST programs (www.nihbest.org). These programs were funded by the National Institutes of Health (NIH) and its Strengthening Biomedical Research Workforce Program to “enhance training opportunities for early career scientists to prepare them for a variety of career options in the dynamic biomedical workforce landscape” (National Institutes of Health 2019). This effort resulted from a June 2012 report from the NIH’s Biomedical Research Workforce Working Group, which revealed that the majority of individuals who earned a biomedical Ph.D. ended up pursuing a career in fields outside of academia, such as in law, industry, government, public service, and other areas. A particular concern of the NIH working group was whether traditional doctoral programs prepared trainees appropriately for a career outside of academe. Because NIH funding is a significant source of support for many graduate trainees (Wilson et al. 2018), the agency’s shift to examine career development signaled a need for institutions to also place a priority on this need.

Over a period of 2 years (2014–2015), NIH awarded non-renewable 5-year grants to 17 applicants that sought to bridge the gap between the traditional, discipline-specific training graduate students and postdocs receive in most doctoral programs, and the training/experience they need for the diversity of jobs they will eventually seek. Of particular relevance is that these grants were specifically experimental: each site was required to collect data that is part of a cross-site evaluation, and NIH has committed to tracking career outcomes to 2032. Each BEST program proposed a novel experimental design, so though we all participated in the same cross-site evaluation, each BEST program differed in its goals, its research design, methods, and programming.

### Data collection

One way that NIH advanced the need for data on this topic was a rigorous cross-site evaluation for all campuses with BEST grants. The MSU BEST program participated in the cross-site data collection strategy developed by NIH and the BEST consortium. To complement this obligation to NIH, MSU used this opportunity to design a data collection strategy that would support research needs we had at our university around participation patterns. As a practical concern, at the end of the grant-funding period, it would be valuable to have MSU-specific data that could be used to compel institutional leaders to allocate institutional resources to continue the program after the non-renewable NIH funding period was over, and capturing participation information would strengthen the evidence surrounding the program’s interventions.

We spoke of participation in BEST activities in terms of dosage (our PI is a Pharmacologist) as a way to measure *participation* patterns, which also provide clues about engagement (Johnson and Stage 2018). To capture data about dosage, we needed a mechanism by which we could gather information such as the *duration* of time spent, the *type* of activity, the *time* when the opportunity is engaged, and other participation patterns that could be instructive. Longitudinally, these data serve as a foundation to answer questions that delve further into how well achieved program learning outcomes and how this impacted career outcomes: are those who get a single, high dosage affected positively in terms of career satisfaction, such as those who might do a three-day, intensive workshop and nothing else? Or is a lower-but-sustained dosage more important so that we might encourage shorter workshops each week to keep a steady level of engagement? What about doses that are not offered by us through our formal programs? What activities consistently are ranked as important by trainees?

But before we could answer the deeper questions about the impact of these experiences or consider the longer-term impacts of experiences, we needed more basic, foundational data about who was participating, the type activity they were participating in, and what frequency. The method by which we sought to answer these questions resulted in the development of the BEST Action Inventory (BAI), an online data tracking system for self-report (Gonyea 2005; Wyse et al. 2014) that could allow participants to capture career-related professional development opportunities. Its development and maintenance was funded by the NIH funding via the MSU BEST program.

BAI is an online data-tracking system of professional development activities for graduate students and postdoctoral scholars engaged in the BEST program that captures the time, duration, frequency and type of activities pursued by participants. The data housed in BAI was protected by the Institutional Review Board of Michigan State University under their Human Subjects protocols.

In the system, trainees could log the number of minutes they spent participating in various professional development activities, including: what type of activity (peer mentoring, workshops, symposia, seminar, etc.); the date of the training opportunity; the number of minutes spent on this activity (minutes, then, being the unit of analysis); and any notes (a field is available where they could make notes for themselves). Time is the unit of analysis (or the dose), with the recognition that time captures participation and not necessarily engagement as we discussed above.

In designing the system, we prepopulated the options with terminology that applied to different activities. We used the terms and categories developed by NIH for the cross-site analysis (Table 1) for activity type. Considering these to be priority activity types, we used the same categories in the BAI system.

**Table 1:** Information provided to participants to help clarify activity types

Activity name	Brief definition (Ask if you're not sure!)
Certification program	A formal certificate program that will appear on your transcript if you complete it. Two common ones for graduate students are the Certification in College Teaching and the Graduate Certificate in Community Engagement
Club	A graduate student organization relevant to your training. Do not include activities such as those affiliated with a religious group or a strictly social group (i.e., sorority or fraternity)
Co-funding source	This would be unusual for you use
Course	This is for a credit-bearing course you might take to build professional skills. A journalism, business, or law class for example
Externship/internship	These are professional experiences where you get to test your professional skills. They can be on campus in a different office (tech transfer, research administration, etc.) or off campus. There is usually a project to them and should not be strictly a research experience
Mixer/networking	An activity where the primary goal is to meet people and network
Peer mentoring	Conversations with peers or other non-professionals about career related issues
Professional mentoring	Conversations with a person whose profession it is to offer you career advice, or getting advice from someone in a job whose counsel you sought (include informational interviews here)
Resource	Books, videos, etc. that you use to inform your professional development
Self-assessment	Birkman Method, StrengthsFinder, DISK or other career-focused assessments
Seminar	Focus is on learning/hearing new information or research. Usually one speaker, and ends with Q&A
Symposium	Akin to a mini-conference, this is an event that can be one or more days, usually features more than one speaker on a single topic. Usually longer than a single seminar, but focused primarily on gaining new knowledge from lectures/talks
Visit to employer site	A "field trip" to visit a workplace
Workshop	A hands-on activity with prescribed learning outcomes, where the goal is for you to gain a new skill (not merely new knowledge)

**Fig. 3:**
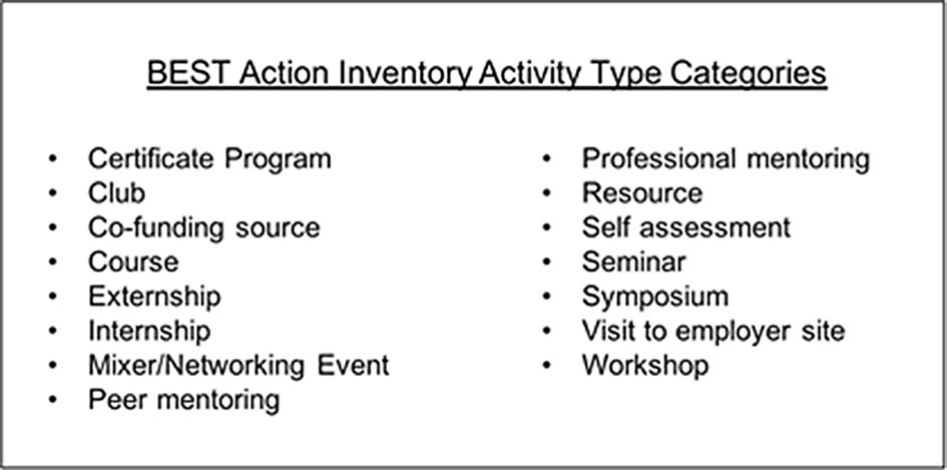
List of activity types in the BEST Action Inventory

Limiting the number of activity types also allowed us to prevent idiosyncratic language choices and helped to ensure consistency for data purposes. For example, one participant may label a career talk as ‘professional mentoring,’ while another would call it a ‘seminar,’ or ‘talk.’ For analytic purposes, we designed the system to constrain the data to limit those variables. Figure 3 describes the types of activities that were pre-populated in the system.

It was important to try to limit variability in the system, so each year at the BEST program orientation we spent a session explaining the BAI system to new participants. We explained the purpose of the BAI system, answered questions about why it was designed the way it was, and demonstrated the kind of data it would capture from each trainee. We reviewed definitions for each “type” of activity (Table 1), so that trainees could select the most appropriate type when they enter in their information. Some categories were easy to discern: “a visit to an employer site” was relatively self-explanatory, a “certificate program” on our campus refers only to official programs that appear on one’s transcript when it has successfully been completed. Less clear for some trainees were distinctions between “workshops” and “seminars,” so we provided definitions to distinguish them. Symposia, on the other hand, were defined for our purposes as akin to a “mini-conference” with multiple speakers around a common topic, that can last one or more days. These tended to be longer than single workshops or seminars.

We trained participants to use BAI in an effort to ensure data integrity. We also built into the BAI design a way to prevent errors and correct errors. System functionality was integrated to pre-populate a BEST activity with date, duration, and type of activity and individual trainees only had to click a single button to affirm attendance. As a routine spot-check, we could compare our analog attendance methods (either sign in sheets or attendance lists kept by program staff, who wrote down who came to BEST activities) with attendance as reported by participants. We could either add them on the back end, or better yet, nudge the trainee and remind them to add their activity to BAI. This helped to build confidence in our data because we could check for consistency between our analog notes and the BAI data.

The possibility to correct data as an administrator was also useful for regular data.

cleaning sessions, where we might find a mislabeled activity type, a doubly entered activity (such as a participant who entered a BEST activity on their own, and then also clicked the system button to affirm attendance), or a mistakenly entered duration of time (such as a workshop that was reported to have lasted from 6 a.m. to 8 p.m. when it should be 6 p.m. to 8 p.m.).

These system checks were also helpful because it provided opportunities for additional education if we saw patterns of mistakes in data entry. We regularly had BAI discussions at BEST events, where we could identify common errors we saw in the data.

The trainees in BEST are all biomedical scientists or engineers themselves, so they had some intuitive appreciation for our need for “clean data.” Further, we echoed NIH’s emphasis that BEST programs were themselves experiments, and we emphasized that trainees engaged in BEST activities were part of that experiment, and students generally brought a certain conscientiousness to their role contributing to the success of the experiment.

After being trained and authorized to use the system, trainees were expected to use BAI to report all of their professional development activities. For official BEST activities, administrators could prepopulate activity data so that attendees could click a single button to log attendance. For non-BEST activities, they would select the activity type, duration, and date. Trainees also had access to a box to input any notes for themselves. From the perspective of educational research, the addition of a notes field encouraged reflection about participation and an opportunity for participants to capture a deeper engagement with the activity.

At any point, trainees could export their personal activities and download an Excel file of their data. The data could then be used by the trainee to update their resume, CV, and individual development plans (IDPs). Additionally, trainees that were requested to fill out NIH progress reports could refer to the data they logged in the BAI to fill out their professional development progress, an important but often neglected component of a training progress report and valuable information for many fellowship program applications. Many such reports ask for participation data, and the BAI tool allows for such data to be captured.

**Fig. 4:**
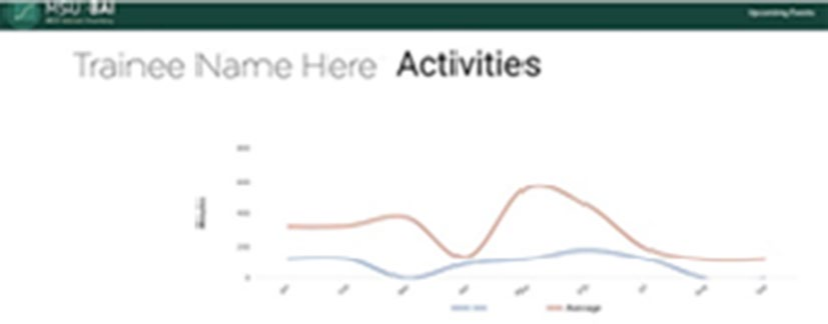
Representative comparative graph of individual/all users in MSU BEST in terms of self activity (blue) and community average activity (red)

Trainees were able to log as much, or as little, data about their activities as they preferred. Some were motivated by comparative measures, so individual users were given a line graph of their activities as an individual over time (in minutes) *vs* the average of all other participants in the system over the same time (Fig. 4). This graph updated in real time as soon as the trainee added an activity, providing feedback that can be gratifying for trainees who like to see visual reminders of their own progress. For people who were motivated by comparing their progress against community averages, this was a useful visual data tracking system. The red line at the top reflects the patterns and aggregate usage of all trainees in the system: the bottom line reflects usage of a user logged into the account. In this example, the selected trainee’s activity was lower, on average, than the group norm.

**Fig. 5:**
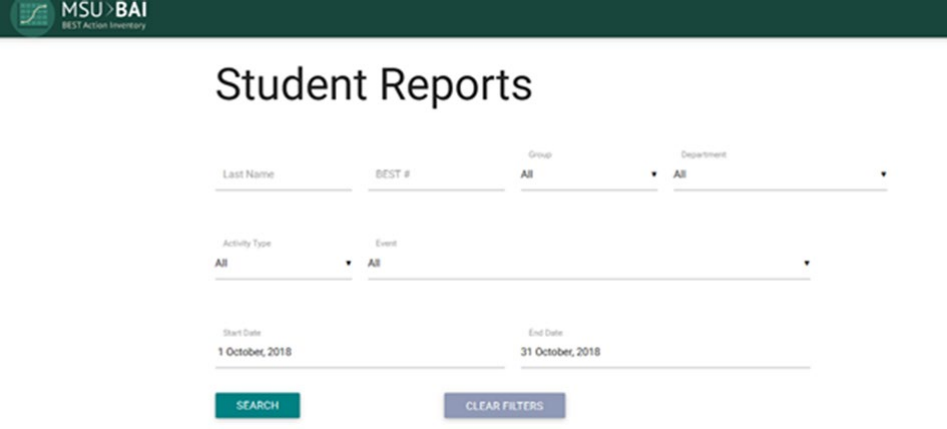
Sample screen illustrating the different fields by which it is possible to search in BAI at the administrative level

Students and system administrators could download activity data at any time. Students could only download their personal record, while administrators could download individual or collective data. It was possible to sort by group (i.e., year in program, departmental affiliation, etc.), status (i.e., early-, mid-, or late-stage graduate school, or post doc), by events or event type (e.g., seminar, workshop, symposia, or other program type.), or by date (Fig. 5).

Each of these reports could be exported and downloaded into an Excel® spreadsheet, which permitted users to analyze the data in different ways and for different purposes without the need for sophisticated software. One use for these data was to make the case to institutional leaders that graduate students and trainees valued programming such as this.

For example, when we approached institutional leaders about the need for continued investment in MSU BEST, there was interest in specific data about participation rates. One campus leader had generally been supportive of helping fund program operations but was compelled to formalize financial support after we showed them that their graduate students and post-docs were participating more often, and in greater numbers, than trainees in similar programs. A demonstrated need, with data comparing their trainees to others, helped make the case for their investment and support.

Another application for these data was program planning. We were able to gather real time data to identify patterns such as which programs were most attractive to trainees, which participants were participating at regular intervals, and other trends. When we developed, scheduled, and offered new activities or training opportunities, we had formal data to support observation data about what, and when, trainees were drawn to professional development opportunities.

### Data analysis

In this section, we share findings from our data. These findings provide useful foundational data for our longitudinal questions about dosage, for program design in providing formative assessment that responds to participant needs, and for building the case to sustain programming. These participation statistics also help support deeper analysis about learning gains and the impact of programming on students and post-docs. These data also enrich the scholarly literature on evidence-based practices in graduate education and training.

For our analysis, we used R, an open-source data analytic software used for statistical computing. This analytic approach allowed us to identify trends in engagement with programming efforts and provided program planners with some clues about how participants were choosing to spend their time in professional development.

**Fig. 6:**
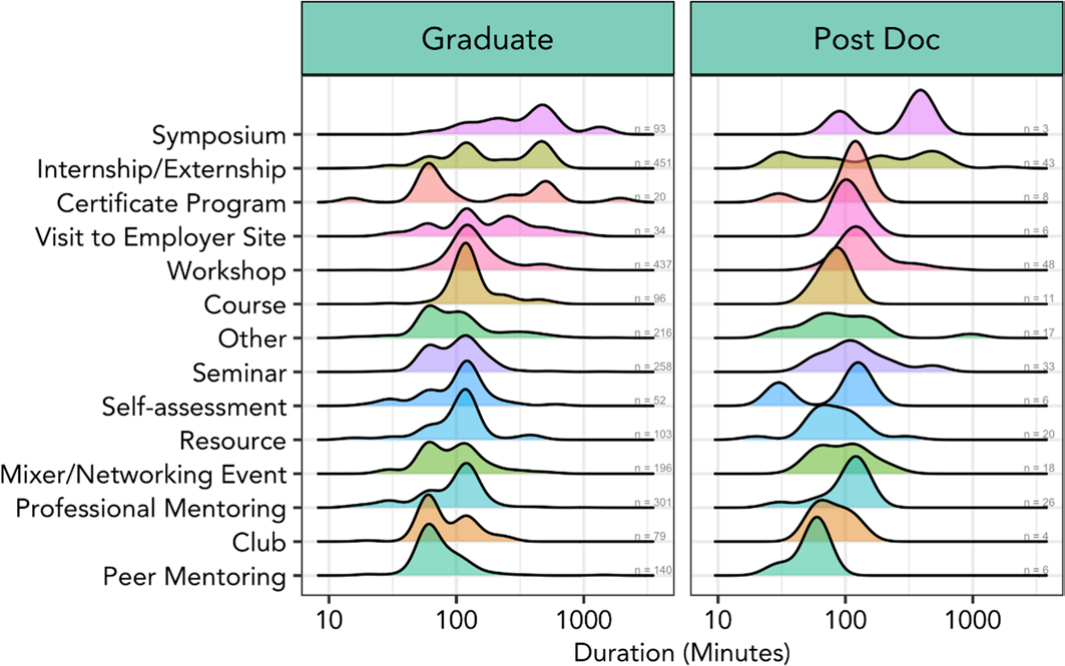
Total minutes in different professional development activity types (2015–2018) for graduate (left column) and postdoctoral (right column) trainees. The *y*-axis lists the type of activity, and the *x*-axis is the duration of the event with the frequency in which it was experienced indicated by the height of colored peaks

Figure 6 illustrates all activities in BAI across four cohorts (*N* = 120). As a collective of trainee participants, these data illustrate that graduate students and postdocs report having spent the most minutes participating in symposia and internships/externships, the latter of which represent practical experiences that provide trainees ‘real world’ experience outside of their traditional training contexts. This is not surprising, as trainees spent a minimum of 40 h in an “internship” or “externship,” and collectively spent the greatest number of minutes engaged in such activities. This finding was useful because we could demonstrate that trainees who participated in internships were not, as some mentors feared, spending inordinate amounts of time “outside the lab”. Rather, we had data to demonstrate that these experiences could be impactful for a few hours a week over a semester or other manageable amounts of times.

As a co-curricular professional development program, MSU BEST program offerings were more likely to focus on workshops (hands-on activities with prescribed learning outcomes), professional mentoring activities (from seasoned professionals who shared their career journeys and offered advice, or from people working as career coaches or counselors), seminars on specific job/skill-related topics, or networking activities. The relatively high participation in “symposia” suggests that trainees engaged in and reported on other professional development activities offered in domains beyond MSU BEST and reflects that symposia tend to be longer events than single workshops or seminars. It also suggests accurate capturing of “symposia” as a category for our system, as symposia were defined for our purposes to be “typically longer than a seminar, focused around a single topic, and often feature multiple speakers.”

**Fig. 7:**
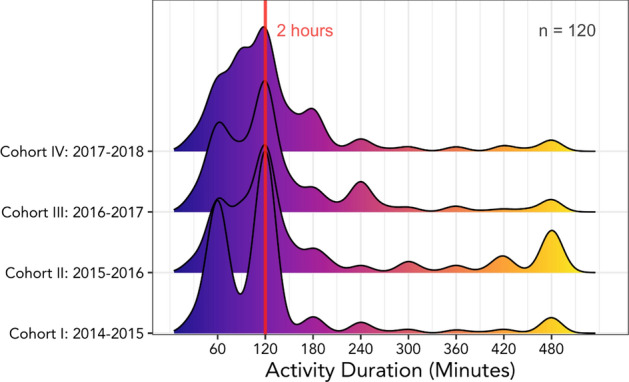
Duration of individual events (across event type, 2014–2018) derived from inputs of graduate and postdoctoral fellows of four (4) cohorts at Michigan State University

Our analysis revealed that most events across type last two hours (Fig. 7). In the early days of our programming, we experimented with different durations for workshops or seminars, but our data supported the observation that participants seemed willing to commit to programs that last two hours; not less, as a shorter program may not have been worth traveling across campus, and not longer, because attention and energy waned. A two-hour target for activities permitted us to start the sessions after traditional work hours and conclude in time for graduate students and post-docs to fulfill their other obligations that evening (i.e., family, homework, experiments). Therefore, our program planners used two hours as a useful target ‘dosage’ for a stand-alone professional development opportunity. That length would not necessarily be suited for every learning program, of course, but it provides useful feedback about what time frames appealed to participants.

Preliminary data reveal that trainees report participating in an average of 15 and 20 total activities over the span of a year. This translates to ~ 2 events per month during the academic year. It is worth noting that there are several trainees who completed a greater number of activities than the average; some report fewer, but 2 events/month reflects the median level of engagement. These data suggest that trainees may self-select into about 4-h/month in professional development programming and find this a reasonable level of commitment that they are able to sustain.

**Fig. 8:**
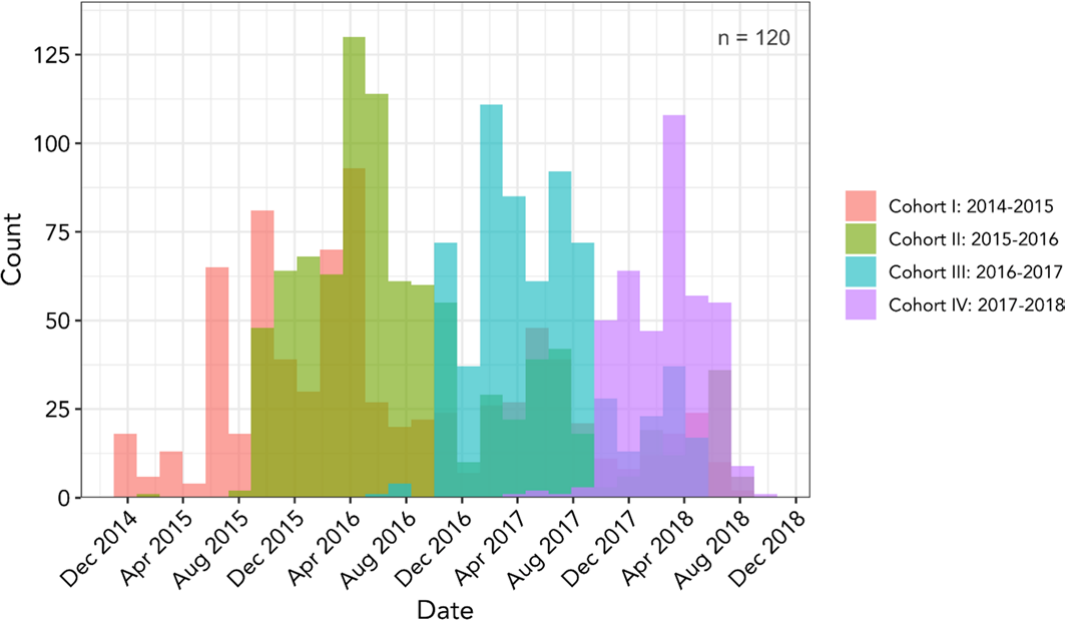
Histogram of the frequency of activity as separated by Cohort. The *Y*-axis depicts the counts of the events, while the *X*-axis bins these activities to different times of the year as experienced by each cohort

We conducted analysis (Fig. 8) with engagement separated by cohort to see if we could glean any patterns about engagement within and across groups. Our program design was such that at the beginning of each academic year, graduate students and post-docs applied to and were selected as a member of a “cohort” (St. Clair et al. 2019). Each cohort followed the curriculum together, participating in events as a group. Each of our cohorts had different demographics (cohorts had different representation of gender, departmental affiliation, and ratio of post docs to graduate students).

The analysis suggests that Cohort II averaged the highest and most enduring participation, though we saw similar behavior patterns in Cohorts III and IV. Cohort I data suggest a lower level of participation, a fact we explain by pointing out that the BAI system was introduced to BEST trainees at the end of their first year in BEST: they had a half-year of BEST programming before the launch of the BAI system. Unlike Cohort I, Cohort II was introduced to the BAI system at the same time as they were oriented to the BEST program and sustained their connection with BEST and with BAI for 3 full years. At this point, our analysis did not identify any statistically significant patterns about participation among members of a particular demographic group, beyond what we noted earlier: that women were overrepresented in BEST relative to the population of women in biomedical science and engineering disciplines, and that international graduate students and post-docs were underrepresented in BEST relative to their presence in the overall target population.

## Discussion

There are numerous ways that we can parse the data in the system, which affords us flexibility in responding to program design, answer preliminary questions about participation patterns, and point us to longitudinal questions that emerge as participants move on in their careers. They have already proven useful in helping narrow down what types of activities, and at what duration students are willing to engage, which is helpful in program design. We used these data to garner support for ongoing program efforts, to engage in conversations with graduate program directors who see a need for graduate students and post-docs but want data to support building something local, or for highlighting the impact we can have on different populations of trainees. There is value, especially for co-curricular options like MSU BEST, to make it as easy as possible for trainees to seek out opportunities, to engage at a level that suits their motivation and goals, in such programs and continue succeeding in their academic program of study.

### Limitations

There are limitations in the system and the data it consolidates. First, there are limits to the value of self-reported data (Gonyea 2005; Pike 2011; Wyse et al. 2014), even though more than half of the articles published in leading higher education journals use self-reported data in their analysis (Pike 2011) and such data are widely used and validated (Porter 2013). This is often in tandem with other sources of data, a trend that is increasing due to advances in mobile technology (Di Fabio and Saklofske 2014; Intille 2012). Many scholars conclude, as we did here, that despite the limits of the data captured via self-report, there are many questions in education that can best be answered by engaging with participants and asking them to capture their own activities and attitudes. We improved confidence in the data by building in functionality within the system to correct errors in user submission, conducting periodic spot checks of data that compare analog data sources with what is found in BAI, restricting what types of activities can be input, and training participants to enter information correctly. Nevertheless, the system relies on participants to enter data and to take ownership of that responsibility.

Though it removed some control from the hands of researchers, there was value in empowering participants to be responsible for the data we collected (with aforementioned safeguards), because it reemphasized that they are responsible for their own professional development. This kind of co-curricular self-directed learning (Knowles 1975) empowers trainees to guide their own development and we believed that self-reporting was a kind of professional responsibility that honored their control over the process. In adult learning, this aligns with best practices in supporting learners in learning contexts outside of formal learning programs.

We also confronted a pragmatic limitation that recognized a need to “start somewhere” and gradually work toward perfecting data collection while trying to minimize lost data and time. Our longitudinal research goal is to connect eventual career outcomes with interventions during graduate and postdoctoral training, to see which “dosage” is impactful. If it turns out that regular, ongoing engagement in professional development opportunities is valuable in the long-term, we needed to find ways to design programming that allows as many students as possible opportunities to participate at that level. Similarly, participation data can also point us to patterns of engagement that we can build support to sustain: If we note that students who participate in activities that imply a deeper level of engagement (i.e., an internship or other sustained effort) achieve their career goals more quickly than those whose participation patterns are limited to potentially passive activities like attending a career panel, that helps guide our programming and focus resources.

We knew that asking participants to be partners in this effort would be key. We missed almost a year of participation data from Cohort I because we were designing and constructing the BAI system in year one of grant funding. We did not want to miss out on more while we tried to overcome every negative scenario caused by self-report data. Thus we engaged in this approach, despite it yielding less than perfect data, because it offered us the best chance to capture complete data (for both BEST activities and non-BEST opportunities) and encouraged active participation in the process from participants from their earliest engagement with the BEST program.

A related limitation of our approach is that the data can be overrepresented by particularly engaged participants and, potentially, inaccurately capture engagement by trainees who have been less diligent about inputting data into the system. An area of attention that we have yet to explore would be to delve further into the qualitative data kept by students under the “notes” option in the system. Future studies could glean useful data about the students who used this feature, and how it shaped their learning. It would also be useful to do a textual analysis of these notes to see if students use them to “summarize” information (who they spoke to, what they said) or as a space for reflective or integrative learning.

There is another important limitation to consider, and that has to do with the population under study, and particularly its focus on only one campus. Given that the work emerged from a BEST Consortium campus, it may seem reasonable to have included data from other BEST campuses for a larger population of participants and for comparative purposes. Studies are forthcoming because each of the 17 campuses involved in BEST participated in the NIH-sanctioned cross-site evaluation process, which had its own research design and variables (the BEST consortium is actively working on comparative analyses from that data set). But not every BEST program embraced the BAI system for a simple reason: each BEST program had its own unique experimental design and not all of them were interested in capturing dosage in the same way, or in tracking individual participant data. Our approach was to track engagement over time to eventually draw conclusions about what “dosage” is most helpful in supporting students with their eventual career goals. This was a unique aspect to our university’s research design, and we were eager to capture both program-specific activities and non-BEST activities. Thus, we encouraged participants to seek out (and log in BAI) a variety of professional development opportunities in varying contexts. Other campuses were focused on different program goals. Therefore, their research question may not have been served well by asking students at every campus to engage with the BAI system because it did not necessarily align with the goals of their campus program.

Despite these considerations, these data contribute to scholarly discussions about the impact and promise of co-curricular programs and are of particular benefit to practical concerns such advocating for institutional support of programs and capturing formative assessment data for use in program design. These limitations point to rich areas for potential research, including replicating this research design on other campuses, optimizing tools like this or Comprehensive Learner Records, and sharing other novel innovations to capture data about student engagement in professional development activities. Mobile technology can innovate the ways we partner with students to capture data about when, where, how, and how often learning experiences take place, and this study shows one example of that.

We also see this kind of tool as being valuable in the ongoing research about engagement and impact of various kinds of educational programming. As we noted earlier, definitions of “engagement” vary, and often focus on educational contexts that have fairly prescribed populations, such as students in an assigned classroom, or focus on educational interventions that are controlled enough to be measured. We hope that this conversation and the introduction of a tool like this enhances this research by establishing the value of capturing data around participation. Like most educators, we are primarily interested in how *our* program affects the students we serve: But we are also open to the idea that our students are served well by a variety of other experiences, other domains, and before we can draw conclusions about the positive effects of our particular program, we were interested in seeing what else participants were doing with their time.

As we have noted throughout this paper, we recognize that a study on participation is both old fashioned and also new. Old fashioned in that it does not go deeper on analyzing engagement, learning, or behavior change; new in that it embraces technology to “start somewhere” to capture data and patterns that have utility in advocating for resources, identifying trends in behavior among students, and exploring ways to understand the fuller landscape of professional development offerings available to graduate students and post docs. We look forward to seeing more scholarly work in this area.

## Conclusion

There are several ways that implementation of a data-tracking system like BAI make a useful contribution to the scholarship on assessment and evaluation in higher education (particularly around program planning and evaluation), on the scholarship of graduate education and professional development, and for education leaders advocating for program support. For example, data from BAI revealed that BEST trainees indicate an average of 2-h spent on activities across types of activity. Many of our activities were, by design, two hours, but the data reveal that students report a 2-h time commitment to be common in other domains. This may suggest that 2-h may be an appealing duration, or a reasonable “ask” from graduate students and postdocs—long enough to find value in what is being offered, but short enough to work in such opportunities around busy schedules.

Research suggests that single one-off workshops may not be impactful on their own (Feldon et al. 2017). In this study, we are not advocating for single, 2-h workshops as the ideal mode of delivery for professional development opportunities so much as suggesting that 2h may be the ideal *length* of a training for busy graduate students and postdoctoral scholars; trainees may prefer activities that are broken up into chunks over a period of time, possibly because they can work that around other obligations in their schedule, as opposed to a longer activity that covers the same ground. As we are looking at these data in terms of dosage, it is potentially as effective to have ongoing “small” doses of intervention as it would be to have one massive dose: the data we report here is a first step in following this idea over time.

As we described earlier, efforts to engage institutional leaders around program support benefitted from concrete data that showed which populations of students were engaging in BEST programs, how often they were participating, and other data that helped us “make our case.” We have also found that for some leaders, the fact of these data being generated by students themselves was more compelling than other strategies such as sign-in sheets or clicker registration because it shows evidence of trainee engagement. Being able to demonstrate that our students were taking advantage of BEST resources, other university programs, and professional development opportunities they created or sought out helped show demand for such training, but also helped point us in direction of what was valued by students beyond what we were already offering. It also helped us identify potential collaborators and guest speakers via students who were logging their non-BEST activities.

In this regard, these results will be useful for scholars and practitioners alike. We offer these findings to the scholarship endeavoring to find “what works” in graduate career development, as a foundation for longitudinal studies of our population, to offer this data tracking system to others interested in exploring its utility (see below) for their own research or campus needs, and as an example of how different types of data sets can be deployed for ongoing research.

This study demonstrates how data collection via the BEST Action Inventory (BAI) data-tracking system can be useful for research purposes as well as program design and assessment, evaluation, and to argue for support of programs from institutional and other funding sources. The design of BAI is particularly valuable for a graduate student or postdoctoral scholar audience because these are populations who are paying increased attention to their career development and preparedness. They are adult learners who can be reliably asked to take ownership of their development and take responsibility for partnering in research like this, and who often need these data for their own purposes, such as annual reviews with mentors or progress reports for funding agencies. These self-report data can be useful to scholars, administrators, and student affairs leaders as well as for the students and postdocs who contribute their time and effort to capture their data within the system.

We share this methodology and these findings to contribute to the conversations being had by scholars and other education leaders about how to develop, assess, study, and foment support for the programs we offer students in higher education. Approaches like this offer data and perspectives with widespread utility, to academic scholars and leaders on a variety of institution types.

## Data Availability

Due to the nature of this research, participants of this study did not agree for their data to be shared publicly, so supporting data is not available.

## Code availability

R was used in the analysis and as an open-access software available at https://www.r-project.org.
